# Academy of Stomatology

**Published:** 1896-08

**Authors:** 


### ACADEMY OF STOMATOLOGY.

THE regular monthly meeting of the Academy of Stomatology was held Tuesday, April 28, 1896. President Dr. S. H. Guilford in the chair.

After the transaction of routine business, the secretary read the proposed changes in the constitution and by-laws; the discussion upon these changes had previously occupied the time of a special meeting called for the purpose. The matter was therefore very briefly considered at this meeting.

Dr. Thomas called attention to the fact that this society had made no contribution to the Horace Wells Memorial fund, although contributions had been received from all over the country, except Chicago, New York, and Philadelphia. It had been left until the last to appeal to Philadelphians, with the hope of securing the largest contribution.

The doctor called attention to the fact that at the next meeting contributions would be asked for that fund. The way other societies had done was by the society contributing what it could, and then the individual members giving what they felt able to give. The speaker said he hoped there would be a large attendance at the next meeting, and that the contributions would be liberal.

Dr. W. L. Mason, of Red Bank, New Jersey, then read a paper on "A System of Detachable Facings."

(For Dr. Mason's paper, see page 488.)

DISCUSSION.

*Dr. Register.*—As I understand Dr. Mason's paper, it strikes me as being quite ingenious. I have given considerable attention to bridge-work, probably being one of the first in Philadelphia to have inserted this kind of work. Some of this is doing service to-day, inserted fifteen years since. I restored a piece not more than ten days ago that had been in use for sixteen years without removal. A number of years ago I was interested in making a crown that was detachable. I made several bridges in which the bridges were placed in position first, and the crowns were put on after the pieces were placed in the mouth. A number of the bridges did quite good service. My method of making the crown was almost opposite to that of Dr. Mason's. Where he makes the dovetail on the posterior in the centre of the crown, I made it on the edges of the crown, and made a septa between each tooth, so that when the work was all finished and soldered together, the crowns were slipped into place with phosphate of zinc.

The objection that I had to that style of bridge was that the division, where it came in between the teeth, was apt to show in the front part of the mouth, and was unsightly. I could not entirely cover that up so as to give a natural appearance to the teeth, so I discarded it. After the all-gold crown came into use, I abandoned the porcelain altogether in all my bridge-work, and used all-gold crowns and dummies, and filled in with vulcanite, so that it is perfectly secure, and nothing but a liquid can get under the dummies. In all these cases there is not an instance I can recall where the patient complained of any disagreeable results. In front teeth I think Dr. Mason's method may be a great improvement.

*Dr. Burchard.*—This subject impresses me as having decided value. Like every method it has its important side, its advantages and disadvantages. What those advantages and disadvantages are, it will take time to discover, but it is decidedly a method worthy of trial. But it seems to me there would be some difficulty in close-bite cases or near-bite cases. Do you find that to be so?

*Dr. Mason.*—No, the troubles you would meet with are more under control than in the old-time bridge.

*Dr. Burchard.*—I must confess I never had any of the trouble in checking porcelain mentioned in the paper, and I would be very sorry to see that branch of technology driven out of existence on account of the lack of skill in using solder. It can be used safely, but for those who have or have not the skill, I think Dr. Mason's method would be of great service.

*Dr. Broomell.*—I would like to demonstrate, if I can, a method I have employed. It is probably, from what Dr. Register says, somewhat similar to the one he adopted some years ago. The dovetail, which I have used in rare cases, is made entirely of metal. I happened to find a tooth in my laboratory to-night with the dovetail in place on the tooth, which I will pass around. I place the back in position first. I make a wedge-shaped dovetail, in this manner. (Indicating on black-board.) This, of course, is a side view; looking on the end of the tooth you have this appearance, that being the labial surface of the tooth, this being the back. (The doctor further illustrates his method by the board.)

The essayist spoke of trouble in checking teeth. I never give that matter any thought, because it seems to me nearly always to be a difficulty that can be overcome.

He also spoke of throwing in borax. I think that is one of the greatest mistakes that can be made. I suppose he means by that that he uses dry borax. That, I think, is what does the damage. The smaller particles of borax will settle down on the investment, whereas, if you use it wet and touch it just where you want the solder to flow, you will overcome any danger of checking.

Another great cause of cracking, I think, is drawing the pins together after the backing is in position. If they are drawn very firmly, it produces a strain, pulling on the porcelain at that point, causing checking from that extra strain put on it by closing these pins together. To overcome this, I think it is policy to barely bend them down sufficiently to keep the back in position, while you apply the solder and heat the piece.

*Dr. Burchard.*—The matter of checking porcelain may not be exactly pertinent to the question, but there is one method that with ordinary care soldering may be almost invariably successful. Porcelain, gold, and solder all have different indexes of contraction. If there is a great amount of solder placed behind the porcelain, in cooling, the porcelain must give way.

(The doctor, then, by illustrations on the black-board, illustrated his method of doing the work.)

*Dr. Kirk.*—I have here a piece of work which is an attempt at removable porcelains, by Dr. M. C. Marshall, I think, of Alabama, presented at the Southern Dental Association, and it may have some features of interest. A box is formed in which this porcelain fits, and the box is then soldered together sufficiently to penetrate between the proximal surface and overlap the buccal surface to make them retentive, and they are then cemented in place.

The device is interesting as one of the methods of securing detachable porcelain, but it seems to me to have an objection in showing a considerable amount of gold. I will pass it around for inspection. This is simply an experimental piece. They are cemented in before the piece is mounted.

*Dr. Mason.*—There seems to be a wrong conception of the dovetail on the back of the tooth. In my paper I said it was not necessary to go into the detailed construction of the tooth, as the teeth would be manufactured for the dentist. I understand some of the gentlemen have an idea that the dovetail is porcelain. It is pure platinum.

*Dr. Truman.*—I would suggest to the essayist that he would do well to alter that part in his paper. It struck me at the time he was reading it that it was not clear. I inferred it was porcelain and wondered how he could saw it off.

*Dr. Guilford.*—Have you made arrangements to manufacture them?

*Dr. Mason.*—I have made very favorable arrangements. I have connected myself with a gentleman very much interested in it, and expect to manufacture the teeth provided we can get the porcelains, and I think we are in a fair way to secure these.

*Dr. Guilford.*—Will each porcelain have a platinum back.

*Dr. Mason.*—The dentist will buy the porcelain with the dovetail soldered in position, also with its back. If you buy a tooth, mould No. 22 to-day, six months from now you can buy a duplicate tooth, lateral, central, or canine.

*Dr. Guilford.*—Do I understand you to say you set them in chloro-percha instead of phosphate of zinc?

*Dr. Mason.*—You can set them in phosphate of zinc if you so choose, but if the porcelain should break off you will have a little more trouble in dislodging the dovetail from its back than where chloro-percha is used. Chloro-percha is firm enough.

The subject was then passed, a vote of thanks being extended to the essayist.

Dr. Jack presented a resolution extending a vote of thanks to Dr. Crouse for his work. After reading the resolution it was unanimously adopted.

Dr. William Truman exhibited to the society a number of books on dentistry, dating from a work written in 1583, in Latin, and extending to an English work in 1839.

The works were submitted for the inspection of the members.

The meeting then adjourned.

---

A meeting of the Academy of Stomatology was held June 23, 1896, at the rooms of the society, for the purpose of discussing the report of the special committee on dental nomenclature of the American Dental Association, the president, Dr. James Truman, in the chair.

Dr. Guilford, the chairman of the committee, had at the May meeting distributed copies of the report to the members of the Academy, in order that searching criticism and comment be made upon the results of the committee's work.

DISCUSSION.

*Dr. Guilford.*—An immense amount of labor has been expended upon this report, and it is by no means in a state of completion. The subject is open to searching criticism by the dental societies here and elsewhere, and by the profession at large. It is hoped through such a general interest we may place our nomenclature upon a sound and rational basis.

I desire to comment briefly upon a few of the features of the report, as it exhibits findings which are at variance with our common views, particularly as relates with the pronunciations of words.

You will notice, first, that the word abnormity is given preferance over abnormality; it is shorter.

The distinction between absorption and resorption is sharply drawn. These words are frequently employed as synonymes, but they are not synonymes. The accent in the word alloy is placed upon the last syllable. Aluminum replaces aluminium. In the word anæsthetic the diphthong *æ* has substituted for it the vowel *e*.

You will notice the plural of apex, which has its accent upon the *a*, is apices, the first syllable being *ap*,—ap′icēs.

Proximate and proximal are preferred to approximate and approximal; the words are then stems upon which any prefixes may be grafted.

The meeting of the masticating surfaces of the upper and lower teeth is frequently spoken of as an articulation; it is more accurately described by the word occlusion.

You will notice that in the word bifurcated the accent is placed upon the second, not the first, syllable.

The words calcareous and calcific are frequently used interchangeably: this is an error. A calcareous substance is one formed of lime; a calcific body is one which forms lime.

A great deal of confusion surrounds the two terms cast and model. Some years ago I inquired of the son of Hiram Powers, the sculptor, the son being a student at the Philadelphia Dental College at the time, what is the distinction sculptors draw between a cast and a model. He answered that a model was something which was copied; a cast was a body formed in a mould. An arbitrary distinction has been suggested, so far as dentistry is concerned, by making model to serve as the title for a plaster cast which is to be reproduced in metal; cast to be applied to the plaster not so reproduced. The distinction is not fully accepted; the practice of calling all plaster casts models appears to be more acceptable.

Of the two pronunciations of cement, cement′ is preferred to cem′ent.

In gutta-percha no reason appears for the *ka* sound in termination; the *ja* is preferred.

Contour is pronounced contour′, not con′tour.

Cor′onal is correct, coro′nal incorrect.

The majority of dictionaries give the preference to dentin over dentine.

In fora′men the accent is upon the *a*′, not upon the *am*′.

The spelling gage has generally displaced gauge.

In the word gingivæ you will note the accent upon a long *i*; it is gin-jī-væ, not gin-givæ.

The word lever is pronounced lev′er, not le′ver.

The singular of matrix is ma′trix, the plural mat′rices.

Pericementum should be the term employed instead of periosteum.

Sixth- and twelfth-year molars are solecisms.

The word solder is omitted from the list; it is to be pronounced sod-der.

Tartar is a word of doubtful origin; it probably arises from the resemblance which the deposits upon teeth bear to the incrustations upon wine-casks.

The words vulcanite and rubber, often used as synonymes, should be each distinguished. Rubber, a soft body of certain composition; vulcanite, a hard body, of a different chemical composition.

The word obturator is restricted to appliances which cover an opening in the hard palate.

A velum is an appliance designed to remedy a deficiency of the soft palate.

The terms sweating and autogenous soldering appear to be unsatisfactory.

*Dr. Burchard.*—In pursuance of Dr. Guilford's suggestion, made at our last meeting, I have examined the dictionary list prepared by the committee of the American Dental Association, and have marked certain parts which I think will bear close discussion. Most of these matters relate to the subject of definitions. It is right and proper that we should dwell upon this matter, for exact definitions are things of no little importance. I am a firm believer in precision, not only as to the application of words, but also to their definitions. Our technical words each should have an individual meaning. Even words which are usually regarded as synonymes may be but partial synonymes; in fact, they usually are, for it is rare that any word has precisely the same meaning as another. These shades of distinction are of importance, particularly from a literary point of view, and it behooves us to make the differentiations precise and clear.

You will notice in the list that the word abnormality is defined as the state or quality of being abnormal; irregularity, deformity, malformation. The word is given as a noun, and you will see that the definition implies, in part, an adjective. Instead of the definition given, it is suggested that one founded upon the etymology of the word would be more precise and explanatory: *An abnormality is an abnormal thing, not state.* "*It is a variation from a general typal form.*"

The next word marked is air-chamber. This is a misnomer; what is defined as an air-chamber is a vacuum-chamber.

It is given as a definition of alloys that they are compounds of metals produced by fusion. This is inexact; *they are compounds formed when the metals are in a state of fusion.* In the verb definition of the same word, to alloy, to reduce the purity of or otherwise modify metals by admixtures, the words *with other metals* should be appended. For the purity of a metal may be reduced without forming an alloy; for instance, the purity of silver is reduced by admixture with sulphur, oxygen, chlorine, bromine, and so on, and yet such compounds, it is needless to state, are not alloys.

The words analgesia and analgesics are omitted. I think a distinction should be drawn between anæsthesia and analgesia. As a rule, we find these words used as synonymes. Anæsthesia is a word which was coined by Dr. Oliver Wendell Holmes to designate the state of insensibility produced through the inhalation of ether or chloroform. It refers to a general abolition of sensation. Analgesia, denotes explicitly an absence of pain; analgesics would therefore include anodynes and obtundents. The root *æsthesis* refers to psychical perceptions,—that is, sensation in the sense that the mind perceives,—therefore the title anæsthetics should be applied to agents which produce general unconsciousness; analgesics to those which destroy sensation, and do not affect general consciousness.

*Dr. Roberts.*—You imply, therefore, that local anæsthetics are analgesics.

*Dr. Burchard.*—Yes; they produce a condition of analgesia, not anæsthesia, as the state described by Dr. Holmes is understood.

For the word anomalous we find the definition: "Deviating from that which is natural." This defines an abnormality, not an anomaly. A definition, to be in consonance with the etymology of the word, should state, "Deviating widely from that which is natural." The same adverb should be inserted in the definition of anomaly.

The word antiodontalgic impresses me as a specimen of needless word-coining.

The definition of asepsis is unsatisfactory and not precise. It states, "Absence of blood-poisoning, exemption from putrefaction and its consequences." The word sepsis should be substituted for putrefactive. These words are not mutually synonymous. Putrefaction always implies sepsis, but sepsis may or may not be due to putrefaction. A comprehensive definition would be, "Asepsis is an absence of those substances the absorption of which causes the condition of septicæmia."

In the definition of antiseptics, septic should be substituted for putrefactive.

In the definition of atavism, the word recurrence is used instead of the usual one, reversion, and it is perhaps better, as it is more precise.

In the definition of the word atrophy, it says, "A gradual wasting of the body." It would be more embracive to say, "A wasting of *a tissue, an organ, or the body*," for atrophy of a single part is very common.

No dental application is implied of the word automatic, and there is no reference to so-called automatic mechanism.

For the definition of asphyxia, the following is submitted, in lieu of that given in the pamphlet: "Asphyxia is a suspension of vital phenomena due to a cessation of normal respiration."

In the definition of calcareous, it is given, "Composed of or containing lime." Calcium salts should be substituted for lime. When we say lime we mean the oxide of calcium; calcareous may or may not refer to the oxide; in fact, it rarely does when used in connection with physiology or pathology.

In the definition of cast, the word matrix should be substituted for mould. A mould is something produced through the operation of moulding; our casts are made in forms which may or may not be moulded, but they are always formed in a matrix.

It is unquestionably correct to say gutta-per-cha, not perka. The former is in consonance with the etymology of the word. The Malay word pertja has been Anglicized into percha.

The word dentalgia is a useless hybrid of Latin and Greek; the word odontalgia, derived from two Greek roots, is certainly preferable.

In defining epithelium, it is spoken of as a "very delicate membrane." This is misleading, for, in point of fact, epithelium represents resistance. Modified epithelial tissue is the most resistant of all tissues. The "very delicate" should be omitted. The following definition is suggested: "Epithelium,—the external cells covering the surface of the body, lining mucous passages and glands."

*Dr. Cryer.*—How about the epithelium of internal structures?

*Dr. Burchard.*—It is a modified epithelium, called endothelium, which lines blood-vessels, the mesentery, etc., parts which are subjected to continuous friction.

In the definition of hypercementosis, precision would suggest a substitution of prepositions. Hypercementosis is exostosis upon, not of, the cementum of the teeth.

*Dr. Truman.*—Why do you draw that distinction?

*Dr. Burchard.*—Because the cementum itself is not the active agent of production. The pericementum deposits cementum upon the existing cementum. The word odontitis is an impossibility; we cannot have inflammation of all parts of a tooth.

*Dr. Cryer.*—Why not?

*Dr. Burchard.*—Unless you subscribe to Dr. Frank Abbot's views as to vital processes in enamel,—and I don't believe you do,—

that tissue and, as we no doubt all believe, the dentine must also be excluded from participation in the inflammatory process.

*Dr. Cryer.*—We say odontalgia in referring to toothache; why not odontitis in connection with dental inflammation?

*Dr. Burchard.*—I think it is a difference of grammatical case, *itis* referring to the genitive case; it is "of." *Algia*, an ablative suffix, in this connection would mean "in."

Odóntoblasts are defined as "the cells which are active in the formation of dentine." Many other cells are also active. The odontoblasts are the cells through which the dentine is formed.

Odontoma is spoken of as "a tumor composed of dentine." Although they do contain dentine, they may also, and usually do, contain other dental tissue, enamel, and cementum.

The peridentium is defined as "the periosteum." The words "of a tooth" should be added, and, even then, why have the word at all? Pericementum denotes explicitly what the peridentium, as here defined, is; besides, the peridentium really is the pericementum and the alveolar process. The word periodontium (not given in the list) is open to the same objection.

Dr. Guilford deplores that the word root, which appears to have no demonstrable logical origin, should be retained. I would suggest one explanation to this matter. First, the root of a tooth is that part implanted in process, and transmits the sources of nutrition. Again, if you recall the appearance of a developing tooth and the process of dentition, its growth is outwardly a resemblance to that of a plant. A root extends downward, the crown comes upward, like the descending and ascending portions of a germinating seed.

Stratum granulosum has a definition which would imply that it is the membrana propria of Garretson. The stratum granulosum is an histological appearance in that portion of the dentine immediately underlying the enamel and cementum.

In the definition of vacuum-chamber, it is stated, "A space entirely devoid of matter." No space can be entirely devoid of matter; the word, as applied, means a space devoid of air.

The word spelt ferrule is advised to be pronounced feril. This seemed so incongruous that I looked up the etymology of the word. It is traced, first, to its French source, *virole*, meaning an iron ring. This is in turn from the middle Latin of *viriola*, a little circlet; it is a derivative of *ferrum*, iron, which is also the etymological source of ferrel. You will notice that the *oo* or the *l* sound is present, but not any sound from which feril could be derived; so that, according to etymology, the pronunciation should be fer-rool or fer-rel.

*Dr. Guilford.*—Nevertheless, the majority of lexicographers give the *il* sound.

*Dr. Burchard.*—Despite that, it is not in accordance with the word's etymology, and, in the absence of recognized rules or reason, why not take the only one available?

In works and discussions upon dentistry, the terms prosthetic and mechanical dentistry are used as mutually synonymous. It appears to me that a distinction should be drawn between them. Prosthetic dentistry includes the planning, devising, and construction of apparatus designed for the replacement of lost dental parts. Mechanical dentistry includes the technical operations through which these appliances are constructed. Prosthetic dentistry, the architect's side; mechanical dentistry relates solely with laboratory technology. Mechanical dentistry is included in the prosthetic, and is but part of it.

The word tartar is commonly believed to have its origin in tartarus, the Greek for hell. The exact significance of the word as applied to wine-cask tartar is given by Paracelsus as follows: the destruction of tartar gives rise to acrid and irritating substances.

*Dr. W. H. Trueman.*—We need only to examine the pamphlet closely to see the amount of painstaking work represented in it. I think the profession at large should be grateful and appreciative to this committee, not only for what they have done, but how they have done it.

